# Retention of Bovie scratch pad radio-opaque marker during VATS Pleurodesis: case report

**DOI:** 10.1186/s13019-021-01497-9

**Published:** 2021-05-05

**Authors:** Deena Akras, Daniel Raymond, Rami Akhrass, Sudish Murthy

**Affiliations:** Department of Thoracic and Cardiovascular Surgery, Heart, Vascular and Thoracic Institute, Cleveland Clinic, 9500 Euclid Avenue, Cleveland, OH 44195 USA

**Keywords:** Pleurodesis, Pneumothorax, Bovie, Scratch pad, Foreign body

## Abstract

**Background:**

Surgical intervention for spontaneous pneumothorax typically includes mechanical pleurodesis that frequently utilizes a Bovie scratch pad given its universal presence, low cost and ease of use. The pad is folded on itself after dividing it in half, allowing easier passage through the smaller incisions.

However, unintentional foreign body retention may occur during the course of an operation leading to reoperations or even worse complications. This case is reported to raise awareness that dividing the scratch pad may allow the embedded radio-opaque marker to fall out and become retained as a foreign body.

**Case presentation:**

The patient is a 41 year-old female who presented with shortness of breath secondary to spontaneous pneumothorax. Chest CT scan showed apical blebs. The patient underwent video assisted thorascopic surgery (VATS) with bleb resection and mechanical pleurodesis using a divided and folded bovie scratch pad. Postoperative chest x-ray showed a retained foreign body. Reoperation confirmed this to be the radio-opaque marker of the scratch pad and was removed. The patient did well and was discharged the following day.

**Conclusion:**

Dividing the bovie scratch pad may damage and “weaken” the product allowing the radio- opaque marker to fall out during its use for pleurodesis and should be discouraged. Recommendation is made of using the scratch pad as a whole and not dividing it.

**Graphical abstract:**

Retained radio-opaque marker of bovie scratch pad during VATS mechanical pleurodesis. 
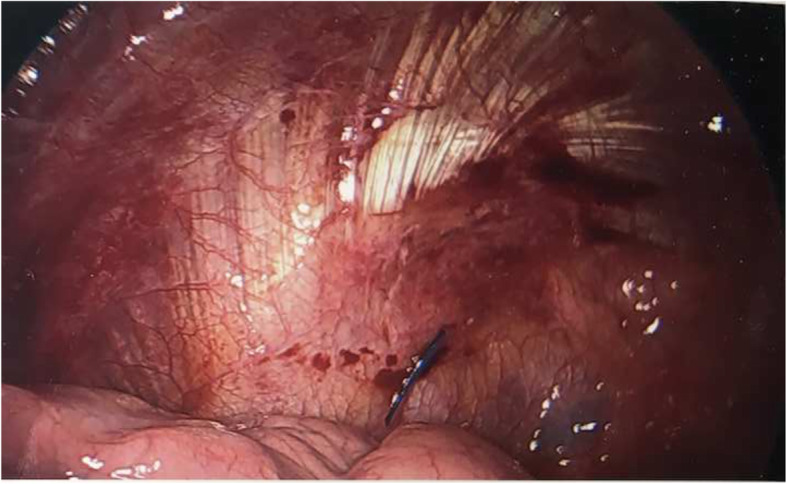

## Background

Spontaneous pneumothorax typically occurs among tall thin adolescent men with an annual incidence of 18–28 and 1.2–6 cases per 100,000 men and women, respectively [1].

It may follow activities that increase intrathoracic pressure but can also occur at rest. Symptoms often include chest pain and/or acute dyspnea, and may resolve spontaneously within 24-h with no intervention [2]. Medical management includes observation for first time smaller pneumothoraces, especially if patients are minimally symptomatic. Tube thoracostomy is usually required in symptomatic patients with larger pneumothoraces.

Video assisted thorascopic surgery (VATS) is performed in cases of recurrences and when blebs are identified radiographically. Surgery usually includes bleb resection in addition to mechanical pleurodesis aimed at promoting adhesions that would prevent lung collapse in case of a recurrence.

It was customary for us to fold the scratch pad on itself after dividing it in half in order to pass it through the smaller VATS incisions. This case is reported to raise awareness that such practice of dividing the scratch pad may damage and weaken the product, allowing the radio-opaque marker to easily separate during its actual use while performing the pleurodesis and become retrained. Foreign body (FB) retention remains the sentinel event most frequently reported to the Joint Commission with 50% occurring in minimally invasive operations. Cardiothoracic surgeries account for 10% of all retained FBs with an incidence of 1 in every 5500 cases [3]. Informed consent was obtained from the patient. Institutional review board approval was not required and was waived for the purpose of this study.

## Case presentation

**Fig. 1:**
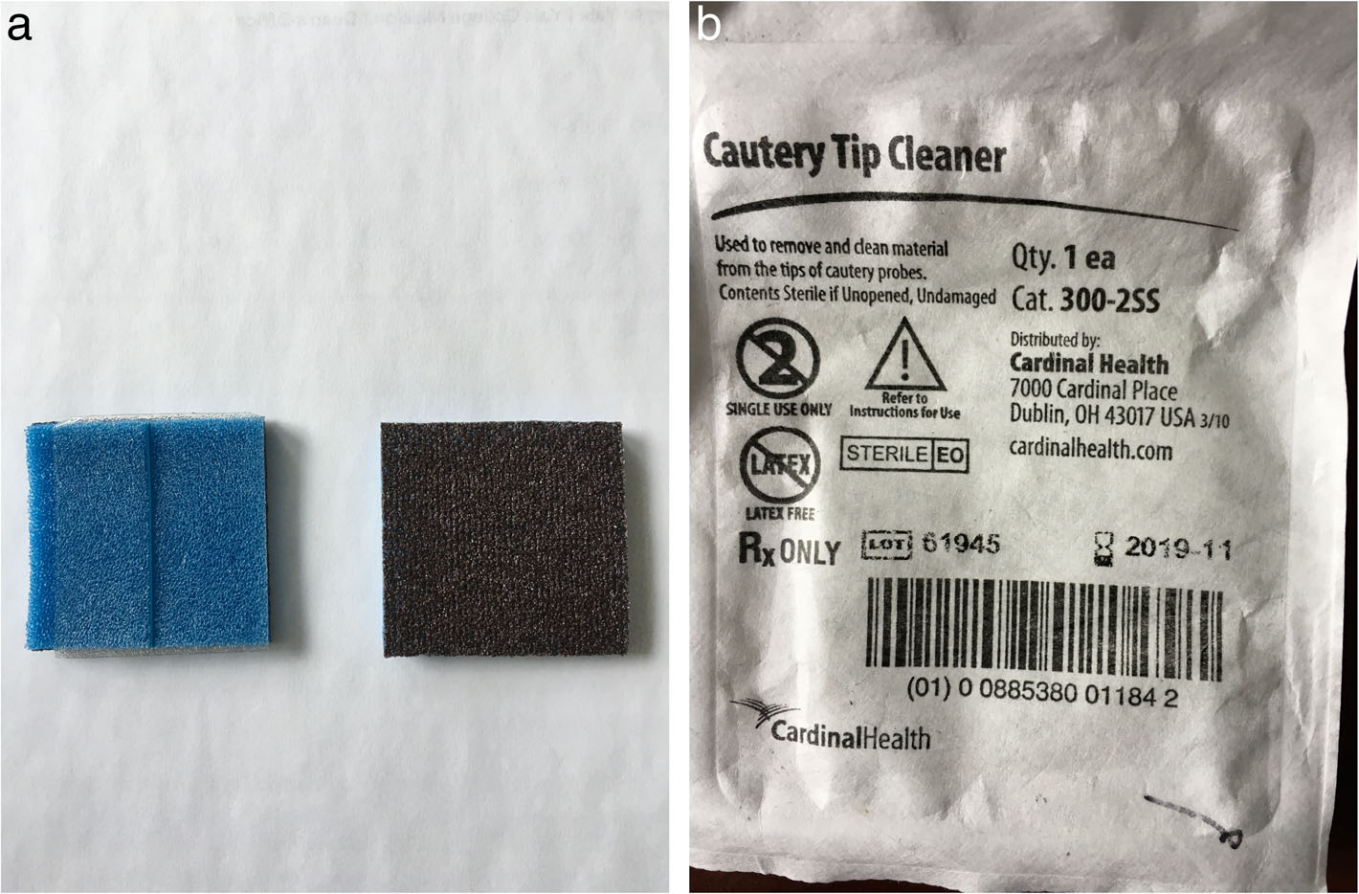
**a** Front of Bovie scratch pad. **b** Back of Bovie scratch pad with central blue radio-opaque marker

**Fig. 2:**
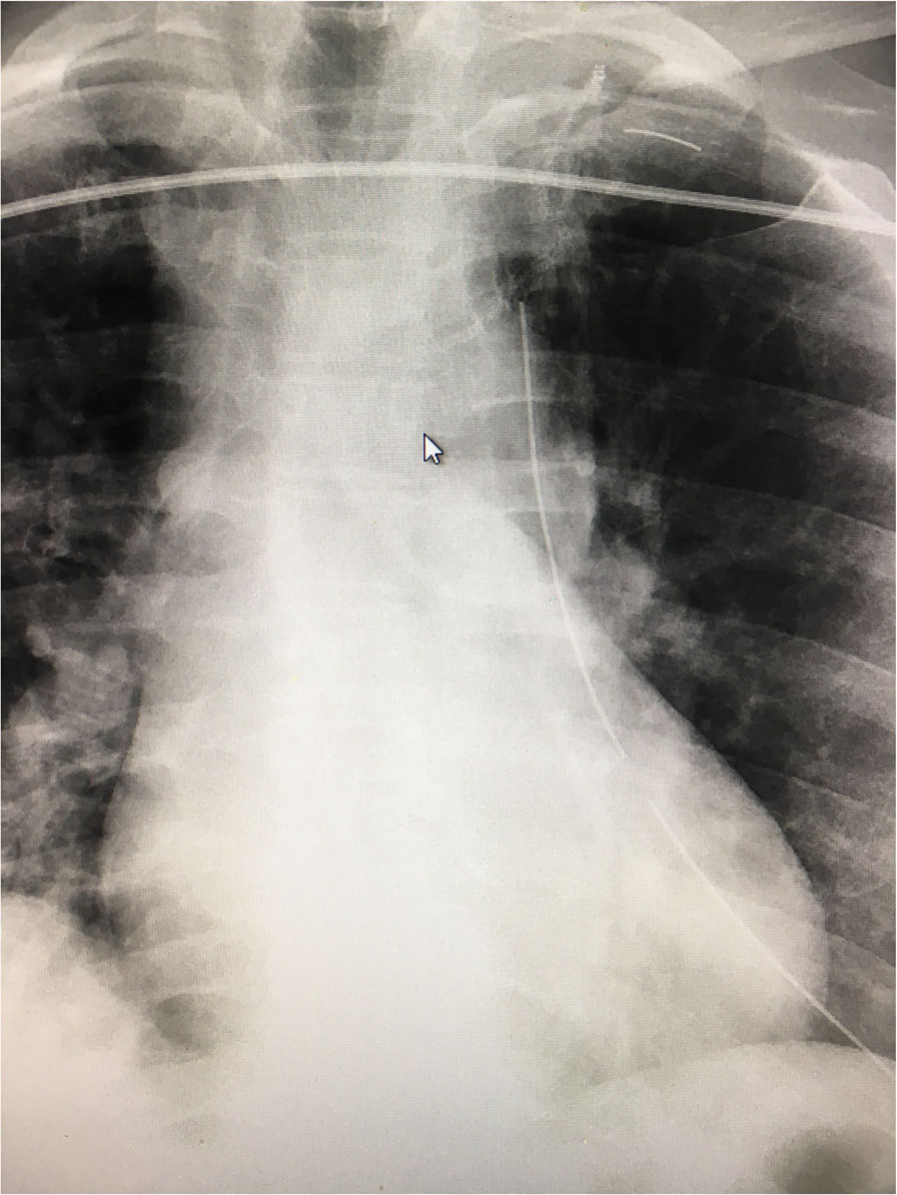
Postop chest x–ray showing radio-opaque marker close to staple line

**Fig. 3:**
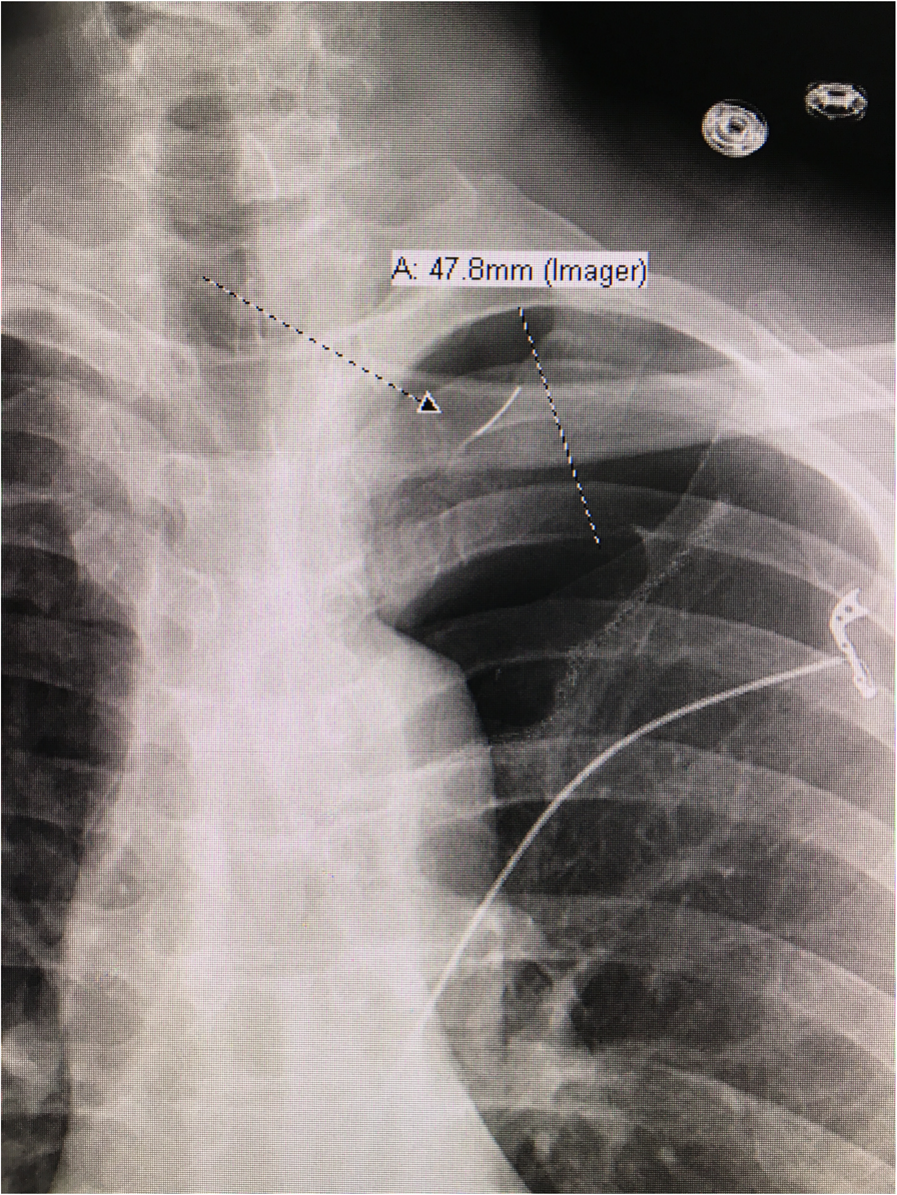
Chest x-ray after chest tube removal with apical pneumothorax and foreign body (FB)

**Fig. 4:**
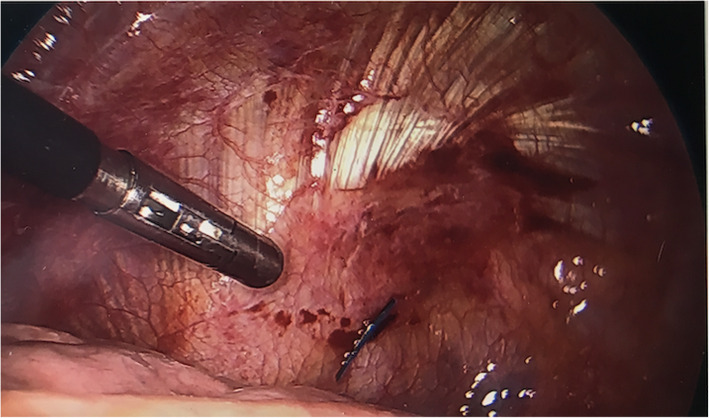
Intraoperative photo with instrument pointing to blue radio-opaque marker in apex of hemithorax

The patient is a 41 year-old female who presented with acute shortness of breath. Chest x-ray and computed tomography showed spontaneous pneumothorax with apical blebs. The patient underwent uneventful Video Assisted Thorascopic Surgery (VATS) with bleb resection and mechanical pleurodesis. Half of a folded Bovie scratch pad that is usually used as a cautery tip cleaner (CardinalHealth, Fig. 1) was utilized to abrade the parietal pleura. The postoperative portable chest x-ray reported no unusual findings (Fig. 2). Following chest tube removal, on postop day-3 prior to discharge, a two-view chest x-ray showed an apical pneumothorax, in addition to an “abnormality” in the apex of the hemithorax (Fig. 3) consistent with a FB. Options, including no intervention versus VATS retrieval of the FB, were discussed with the patient who opted for the latter. Intraoperatively, the FB was easily identified and removed (Fig. 4). The patient was discharged the following day in good condition. The FB was confirmed to be the radio-opaque marker of the Bovie scratch pad.

## Discussion

Surgical intervention for spontaneous pneumothorax is recommended for blebs identified on chest CT-scans, especially in cases of recurrence or in patients who might engage in “high-risk situations”, such as flying, scuba diving or hiking, where access to medical care may not be immediately available. Surgery is typically performed through VATS approach that includes bleb resection and pleurodesis, usually mechanical, aimed at preventing lung collapse and tension pneumothorax in case of a recurrence by creating adhesions between the parietal and visceral pleura [4, 5].

Mechanical pleurodesis is frequently accomplished by using the Bovie scratch pad, an off-label application (Fig. 1). It is intended to clean the electro-cautery tip when needed; however, its universal presence, low cost and ease of use have led many thoracic surgeons to use them for pleurodesis. Its use has been reported by others and described in the training manual of the Society of Thoracic Surgery (STS) [6]. We have successfully carried out this practice for many years without any adverse effects.

The instrument count at the conclusion of the operation was correct, as the counted item was the pad itself and not the marker. The radiological report of the immediate portable postop chest x-ray did not mention the FB, although in retrospect it can be visualized. The marker was in close proximity to the parenchymal staple line and thought to be part of it (Fig. 2). It became more apparent when an apical pneumothorax, following chest tube removal on day three, moved the staple line away from the marker (Fig. 3). Our index of suspicion was high that the abnormality seen was the marker but could not be absolutely certain, as the pad had been already discarded as one might expect.

It was formerly customary for us to cut the scratch pad in half for easier introduction through the small VATS incision. This perhaps “weakened” the product and permitted the marker to separate from the pad, as it is difficult to remove it from an intact and uncut pad. One thought was to remove the marker off the cut pad prior to its insertion into the chest. This perhaps may create a worse problem of retaining an unmarked pad that cannot be detected radiographically in case of an incorrect count. It is also against our and probably many others’ institutional policies of minimizing use of unmarked instruments and devices.

Unintentional FB retention is a dreaded complication that all involved in the care of patients strive to eliminate. Our current practice is to continue to perform pleurodesis per STS guidelines, unless further studies corroborate the work by Min and colleagues showing no difference in recurrence rates in patients with and without pleurodesis [7]. The Bovie pad is used as a full (uncut) pad or can be trimmed from sides but parallel to the marker and not across it. A conscious effort is also made at the end of each case to specifically identify the marker within the scratch pad at time of instrument count.

## Data Availability

All data generated or analyzed are included in this published article.

## Abbreviations

FB: Foreign body
STS: Society of thoracic surgery
VATS: Video assisted thorascopic surgery
